# Assessment of Community Levels of Knowledge About Developmental Dysplasia of the Hip, its Risk Factors, Treatment, and Complications in the Riyadh Region, Saudi Arabia

**DOI:** 10.7759/cureus.30465

**Published:** 2022-10-19

**Authors:** Mohammed J Alanazi, Wejdan Abokhesheim, Raneem M Al Saqer, Reem Alasmari, Razan M Alotaibi

**Affiliations:** 1 Division of Orthopedics, Department of Surgery, King Abdullah Bin Abdulaziz University Hospital, Riyadh, SAU; 2 Department of Orthopaedics, Princess Nourah Bint Abdulrahman University, Riyadh, SAU; 3 College of Medicine, Princess Nourah Bint Abdulrahman University, Riyadh, SAU

**Keywords:** ksa, saudi arabia, riyadh, complication, treatment, risk factors, knowledge, awareness, developmental dysplasia of the hip, ddh

## Abstract

Background

Developmental dysplasia of the hip (DDH) can involve an immature hip, acetabular dysplasia with or without subluxation, or dislocation of the femoral head. The prevalence of DDH in Saudi Arabia is 10.46 per 1000 live births, highlighting the importance of community knowledge of DDH risk factors and treatment to facilitate early diagnosis, treatment, and avoiding complications.

Aim

Our goal is to assess community awareness of DDH and the knowledge of its risk factors, treatment, and complications among the population in the Riyadh Region, Saudi Arabia.

Methods

A cross-sectional study on a sample of 412 participants of the general population in the region of Riyadh, Saudi Arabia was conducted using the snowball sampling technique of sending invitations via social media. All statistical analyses were conducted using SPSS Statistics v.23 (IBM Corp., Armonk, NY).

Results

Our results revealed that out of the 412 participants almost half reported never hearing about DDH (45.6%). Breech presentation, family history, and female gender were unknown as risk factors to 63%, 58%, and 63.60% of participants respectively. Around 60% of the participant viewed age as the determining factor for DDH treatment. Additionally, 42.70% of participants reported no knowledge about DDH complications, and 72.8% had a low knowledge level regarding DDH. Significant higher knowledge scores were found in females participants (p = 0.026), participants with higher education level (p = 0.01), healthcare professionals (p < 0.001), parents of children who have been screened (p < 0.001), and participants having a first-degree relative with DDH (p < 0.001).

Conclusion

Our study revealed that residents of the Riyadh Region were unaware of DDH and their knowledge regarding its risk factors, treatment, and complications was poor. Therefore, it is important to implement DDH awareness campaigns to increase the community's knowledge of all aspects of DDH.

## Introduction

Developmental dysplasia of the hip (DDH), previously known as congenital dislocation of the hip (CDH), is a spectrum of hip abnormalities in infants and children. DDH is defined as an immature hip, acetabular dysplasia with or without subluxation, or dislocation of the femoral head [1]. Numerous DDH risk factors include breech position, female gender, firstborn, family history, kinship, and swaddling [2,3]. The screening for DDH includes a physical examination, mostly used universally, with hip ultrasound in some countries [1]. But these methods are controversial as 15% of cases are not detected by examination or ultrasound at birth. The potential short and long-term complications of untreated DDH range from leg length discrepancy or waddling gait to hip arthritis in early adulthood [1,4]. This highlights the importance of DDH awareness and screening to help in early detection. DDH management ranges from conservative management using Pavlik harness or closed reduction and hip spica in younger patients to surgical open reduction and pelvic osteotomy in older patients, with or without femoral shortening osteotomy [4].

The prevalence of DDH in Saudi Arabia is 10.46 per 1000 live births [3]. A study done at a tertiary hospital in Riyadh showed that DDH is more common among females, with less than half of DDH patients having bilateral disease [5]. According to another study in Al-Madinah, Saudi Arabia, most cases were females, and two-thirds of the infants with DDH had unilateral hip dysplasia [6]. Moreover, a study held in Arar city, Saudi Arabia, showed that 70% of the affected children had a positive family history [4]. A systematic review of Saudi literature showed consanguinity as a risk factor in 32.9% of patients [3].

Additionally, the Saudi population had a genetic link between GDF5 (SNP rs143383) and DDH. Furthermore, the genotype TT and the T allele were over-expressed in the patients and their fathers [7]. Regarding the awareness of DDH among parents in Riyadh, a study showed that parents were relatively unaware of DDH [8]. Furthermore, the awareness of the correct method for swaddling and the harmful effects of poor swaddling techniques was substandard among females in Saudi Arabia [9]. Additionally, the diagnosis time was very late; 88.8% of the cases were diagnosed after three months [5]. In addition, neglected DDH cases treated with surgery account for around 30% of the surgical practice of most pediatric orthopedic surgeons in Saudi Arabia [10].

Knowing the risk factors and suspicion of DDH allows people to screen, detect, and treat this condition earlier to avoid potential risks and complications with late treatment. While different studies have focused on DDH risk factors worldwide, there has been a lack of data on the awareness of DDH and its risk factors among the Saudi population. In other words, raising awareness and educating the public will greatly assist them in detecting, screening, and seeking early care. Since early diagnosis and treatment are crucial for the prognosis of the disease, this study aims to assess the awareness of DDH and the knowledge of its risk factors, treatment, and complications among the population in the Riyadh Region, Saudi Arabia.

## Materials and methods

Study design and population

This cross-sectional study was conducted through an Arabic self-reported online questionnaire from October 30th, 2021, to January 24th, 2022. The questionnaire was distributed among residents of the region of Riyadh aged 18 years and older. Consent was obtained from all participants before enrollment in the study.

Data collection tool

The questionnaire contained five sections. The first section was demographics, including age, gender, marital status, nationality, level of education, profession, and relatives with DDH. The second section was the DDH awareness level; it included questions to review previous knowledge, source of information, and the reliability of knowledge with general questions about DDH. The third section was about DDH risk factors, the fourth was about the treatment, and the fifth was about the complications. In these sections, the knowledge level of the respondent on DDH was estimated with multiple answer questions, which included direct and indirect questions with correct and incorrect answers. These questions and answers were referenced from orthopedic literature and revised by a pediatric orthopedic clinician. The questionnaire was conducted through a pilot test, followed by face validity by sending the survey to a pediatric orthopedic surgeon.

Sample size

The sample size was calculated using Cochran's formula based on a previous study [8], with a margin of error = 0.05, proportion of population with the attribute = 68.8%, and Z score = 1.96 (Za = 0.95, p = 68.8%, d = 0.05). The minimum sample size was 330.

Statistical analysis

Data analysis was performed using SPSS Statistics for Windows, v. 23 (IBM Corp., Armonk, NY). Frequency and percentages were used to display categorical variables. Minimum, maximum, mean, and standard deviation were used to present numerical variables. Independent t-tests and ANOVA tests were used to test for association. The ANOVA test was followed by Tukey’s post-hoc test to determine where the exact significant difference between the groups exists. The level of significance was set at 0.05.

Ethical consideration

This study was conducted and approved on October 3, 2021, by the PNU Institutional Review Board (IRB Log#21-0369).

## Results

A total of 412 participants were included in the study. The sociodemographic profile of the participants is shown in Table 1. Among the participants, 129 (31.3%) were between 26 and 35 years old. Most participants (290, 70.4%) were females, 408 (99%) were of Saudi nationality, and 265 (64.3%) were married. More than half (238, 57.8%) had a bachelor’s degree, and most (340, 82.5%) were non-healthcare professionals. There were 225 (54.6%) who reported having children.

**Table 1:** Socio-Demographic Profile of The Participants  (n = 412)

Demographic charactaristics		n	%
Age			
18-25 years		107	26.00
26-35 years		129	31.30
36-45 years		100	24.30
Over 45 years		76	18.40
Gender			
Male		122	29.60
Female		290	70.40
Nationality			
Saudi		408	99.00
Non-Saudi		4	1.00
Marital Status			
Single		147	35.70
Married		265	64.30
Education level			
Intermediate school and less		16	3.90
High school		123	29.90
Bachelor’s degree		238	57.80
Higher education		35	8.50
Profession			
Healthcare professional		72	17.50
Non-healthcare professional		340	82.50
Do you have children?			
Yes		225	54.60
No		187	45.40

The participants’ previous exposure to DDH is displayed in Table 2. As regards their familiarity with DDH, 224 (54.4%) were familiar with it. Moreover, 126 (30.6%) of parents reported that their children were screened for DDH, while 99 (24%) were not, and the remaining 187 (45.4%) reported not being a parent. In addition, 55 (13.3%) reported having a first-degree relative (sister, brother, son, or daughter) with DDH. Besides, 104 (25.2%) reported knowing someone with DDH.

**Table 2:** Questions Regarding Participants' Previous Exposure to Developmental Dysplasia of the Hip

Question		n	%
Have you ever heard about developmental dysplasia of the hip?			
Yes		224	54.4
No		188	45.6
If you have children, have they been screened for developmental dysplasia of the hip?			
Yes		126	30.6
No		99	24
I don't have children		187	45.4
Do you have a first-degree relative (sister, son, daughter, brother, sister) with DDH (developmental dysplasia of the hip)			
Yes		55	13.3
No		357	86.7
Do you know anyone with developmental dysplasia of the hip?			
Yes		104	25.20
No		114	27.70
I'm not sure		194	47.10

**Figure 1:**
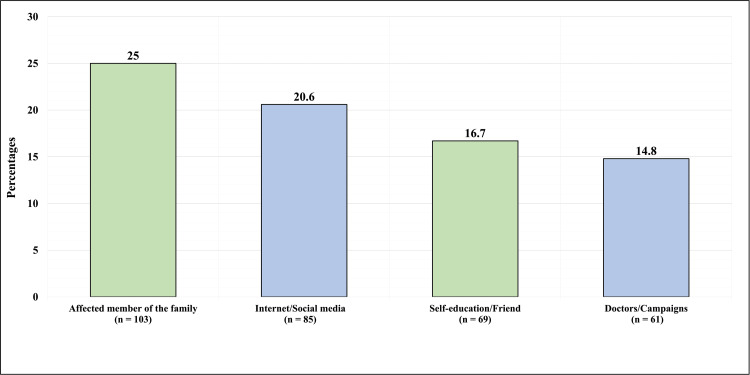
Participants' Source of Information about Developmental Dysplasia of the Hip

Figure 1 shows the participants’ source of information about DDH. The majority of participants, 103 (25%), reported knowing about DDH from an affected family member. At the same time, 85 (20.6%) reported knowing DDH from the internet/social media. Sixty-nine (16.7%) reported knowing of DDH from self-education/friends, and 61 (14.8%) reported knowing DDH from doctors/campaigns.

Tables 3-4 report the assessment of the participant’s awareness and knowledge of DDH risk factors, treatments, and complications.

**Table 3:** Assessment of Participants' Awareness Regarding Developmental Dysplasia of the Hip and its Risk Factors

Question	n	%
Which one of the following best describes developmental dysplasia of the hip?		
A hip disorder, allowing the hip joint to become partially or completely dislocated (DDH) (correct answer)	129	31.3
The head of the thighbone slips with respect to the rest of the bone (SCFE)	193	46.8
I don't know	90	21.8
When should a person with DDH get diagnosed?		
Since birth to 6 months (correct answer)	182	44.2
6 months to 1 year	33	8
1 year	15	3.6
2 years	6	1.
I don’t know	176	42.7
Developmental dysplasia of the hip commonly occurs in premature birth babies.		
Yes (correct answer)	73	17.7
No	80	19.4
I don't know	259	62.9
Developmental dysplasia of the hip commonly occurs in babies with breech presentation.		
Yes (correct answer)	124	30.1
No	28	6.8
I don't know	260	63.1
Developmental dysplasia of the hip is most common in girls.		
Yes (correct answer)	89	21.6
No	60	14.6
I don't know	263	63.8
Swaddling can lead to dislocation of the hip joint.		
Yes (correct answer)	62	15
No	129	31.3
I don't know	221	53.6
Males and females are equally affected by developmental dysplasia of the hip.		
Yes	126	30.6
No (correct answer)	60	14.6
I don't know	226	54.9
A firstborn child has a higher risk of developing developmental dysplasia of the hip.		
Yes (correct answer)	75	18.2
No	83	20.1
I don't know	254	61.7
During pregnancy, if the fluid around the fetus is low in amount, developmental dysplasia of the hip can develop.		
Yes (correct answer)	59	14.3
No	46	11.2
I don't know	307	74.5
Family history of developmental dysplasia of the hip can contribute to developing a new case of developmental dysplasia of the hip.		
Yes (correct answer)	89	21.6
No	81	19.7
I don't know	242	58.7
Developmental dysplasia of the hip can occur in both hips.		
Yes (correct answer)	137	33.3
No	52	12.6
I don't know	223	54.1
Developmental dysplasia of the hip is highly related to the intrauterine position of the baby.		
Yes (correct answer)	113	27.4
No	48	11.7
I don't know	251	60.9
Extrauterine environment can play a role in developmental dysplasia of the hip.		
Yes (correct answer)	135	32.8
No	45	10.9
I don't know	232	56.3
A high birth weight baby can cause developmental dysplasia of the hip.		
Yes (correct answer)	102	24.8
No	62	15
I don't know	248	60.2

**Table 4:** Assessment of Participants' Knowledge Regarding the Treatment and Complications of Developmental Dysplasia of the Hip

Question	n	%
What do you think the treatment from birth to 6 months?		
Observe the child	67	16.3
Physiotherapy	124	30.1
Conservative (Pavlik harness and abduction splint) (correct answer)	164	39.8
Surgical correction (Closed Vs open reduction)	57	13.8
What do you think the treatment from 6 months to 18 months?		
Observe the child	38	9.2
Physiotherapy	120	29.1
Conservative (Pavlik harness and abduction splint)	172	41.7
Surgical correction (Closed vs open reduction) (correct answer)	82	19.9
What do you think the treatment from 18 months and older?		
Observe the child	33	8
Physiotherapy	97	23.5
Conservative (Pavlik harness and abduction splint)	76	18.4
Surgical correction (Closed vs open reduction) (correct answer)	206	50
When do you think is the best time to treat developmental dysplasia of the hip?		
As soon as possible (correct answer)	265	64.3
When the child walks	88	21.4
When the child hit puberty	16	3.9
DDH doesn’t need any treatment	43	10.4
According to the previous question, why did you choose the time?		
To prevent complications of developmental dysplasia of the hip if left untreated	234	56.8
Give the child chance to grow	95	23.1
To prevent a more invasive management	79	19.2
Other	4	1
Should all the cases of developmental dysplasia of the hip get treatment?		
Yes (correct answer)	194	47.1
No	19	4.6
Depends	196	47.6
I don't know	3	0.7
Do you think developmental dysplasia of the hip causes complications?		
Yes (correct answer)	215	52.2
No	21	5.1
I don't know	176	42.7

**Figure 2:**
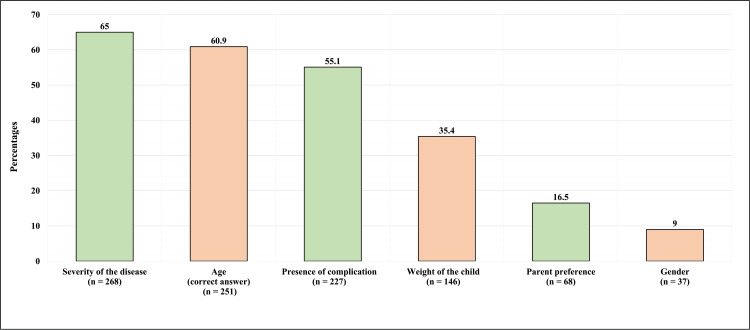
Participants' Thoughts Regarding What Determines the Management of Developmental Dysplasia of the Hip

Regarding the participants’ thoughts on what determines the management of DDH, only 251 (60.9%) correctly identified age as the determining factor. The most common misconception about DDH management was considering the severity of the disease to be the determinant in 268 (65%), whereas 227 (55.1%) considered that complications determine the management, and 146 (35.4%) believed that the child’s weight is the determinant (Figure 2).

**Figure 3:**
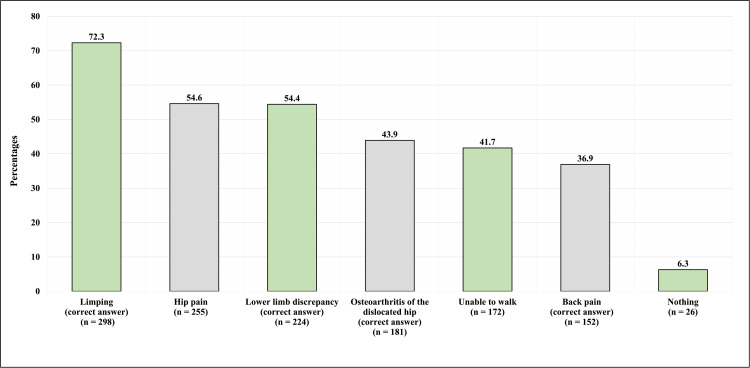
Participants' Thoughts Regarding The Complications of Developmental Dysplasia of the Hip if Left Untreated

Figure 3 shows the participants’ thoughts toward the complications of DDH if left untreated. Most (298, 72.3%) correctly identified limping as a complication. In addition, 255 (54.6%) have correctly identified hip pain as a complication. Also, 224 (54.4%) have correctly identified lower limb discrepancy as a complication, and 181 (43.9%) have correctly identified osteoarthritis of the dislocated hip as a complication. Less than half (172, 41.7%) identified untreated DDH as responsible for an inability to walk, and 152 (36.9%) correctly identified back pain as a complication. Unfortunately, 26 (6.3%) participants believe that there are no complications for DDH if left untreated.

In addition, the knowledge score of DDH. The minimum score was 0, the maximum was 24, and the mean was 8.89 ± 5.4.

**Figure 4:**
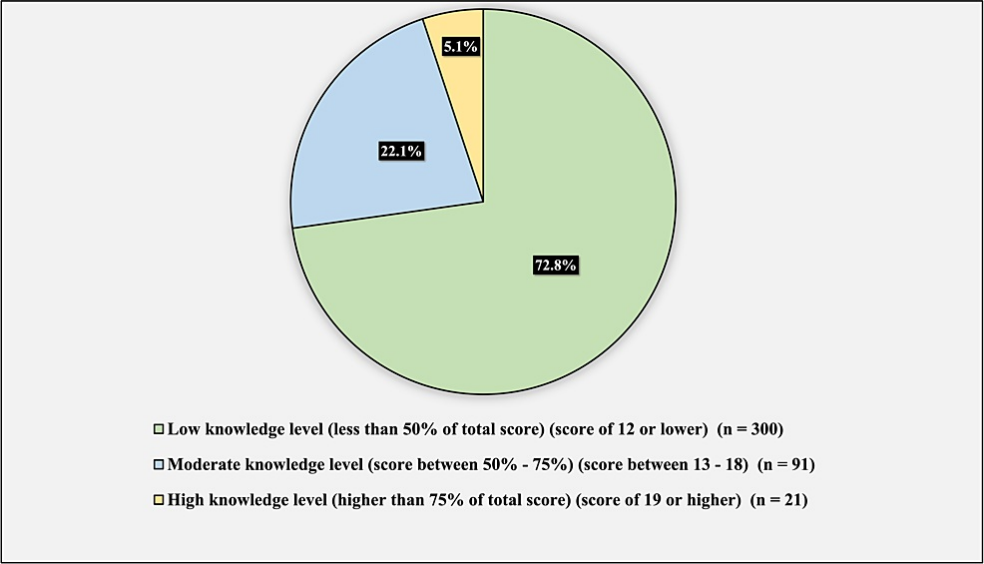
Knowledge Level Regarding Developmental Dysplasia of the Hip

Figure 4 represents the knowledge levels of DDH. The majority of our sample, 300 (72.8%), had a low knowledge level (less than 50% of the total score) (score of 12 and less). And 91 (22.1%) had a moderate knowledge level (score between 50-75%) (score, 13-18). Twenty-one (5.1%) had a high knowledge level (higher than 75% of the total score) (score of 19 and high).

Table 5 demonstrates the factors associated with DDH knowledge. Gender was significantly associated with DDH knowledge (p = 0.026), where it was observed that females had a significantly higher knowledge score compared to males (9.27 ± 5.23 vs. 7.98 ± 5.7). Education level was also significantly associated with a higher knowledge score toward DDH (p = 0.01), where it was observed that the higher the education level, the higher the knowledge score. Tukey’s post-hoc test did not reveal a significant difference when comparing each pair of education levels. The profession was also significantly associated with knowledge score toward DDH, where it was observed that healthcare professionals had a significantly higher knowledge score compared to non-health-care professionals (p < 0.001). Parents who reported having a child screened for DDH had a significantly higher knowledge score compared to the parents who did not have a child screened for DDH (p < 0.001) (10.87 ± 4.87 vs. 6.92 ± 4.47). Having a first-degree relative with DDH was significantly associated with a knowledge score toward DDH (p < 0.001), where it was observed that those with a first relative with DDH had a higher knowledge score compared to those with no first relative with DDH (13.02 ± 4.13 vs. 8.25 ± 5.29). Age, nationality, marital status, and having children were not significantly associated with knowledge of DDH.

**Table 5:** Factors Associated With Knowledge Toward Developmental Dysplasia of the Hip *Significant at level 0.05

Factor		Knowledge Score		P-Value
		Mean	Standard deviation	
Age				0.398
18-25 years		9.35	5.66	
26-35 years		8.66	5.76	
36-45 years		9.26	5.20	
Over 45 years		8.13	4.58	
Gender				0.026*
Male		7.98	5.70	
Female		9.27	5.23	
Nationality				0.070
Saudi		8.84	5.39	
Non-Saudi		13.75	4.57	
Marital Status				0.484
Single		9.14	5.95	
Married		8.75	5.08	
Education level				0.01*
Intermediate school and less		6.56	6.24	
High school		7.89	5.12	
Bachelor’s degree		9.36	5.21	
Higher education		10.23	6.52	
Profession				< 0.001*
Health care professional		11.83	6.09	
Non-health care professional		8.26	5.03	
Do you have children?				0.317
Yes		9.13	5.08	
No		8.59	5.76	
If you have children, have they been screened for developmental dysplasia of the hip?				< 0.001*
Yes		10.87	4.87	
No		6.92	4.47	
Do you have a first degree relative (sister, son, daughter, brother, sister) with DDH (developmental dysplasia of the hip)				< 0.001*
Yes		13.02	4.13	
No		8.25	5.29	

## Discussion

Regarding the public familiarity with the term DDH, nearly half of our sample had never heard of DDH. People with DDH already knew about it because they know an affected person, either a first-degree relative or someone else, accounting for a quarter of our participants. This correlated with a study that stated that people were knowledgeable about DDH because they have an affected family member [8]. However, a minority of our participants had previous exposure to DDH through doctors and educational campaigns. Indeed, it is unfortunate that people did not receive sufficient education from healthcare providers regarding this issue. Similarly, a study assessing parents’ knowledge about DDH revealed that only 6.8% grasped their DDH information from doctors [8]. Most studies in Saudi Arabia suggest implementing a national program to detect and screen DDH, yet it is evident that such programs do not exist [5,8,9,11]. Therefore, we suggest that increasing public awareness of DDH will positively influence the progression and improvement of the provided healthcare for the child.

In previous studies, DDH has been recognized to be a condition that progresses over time and develops during the period before and even after birth [8]. As for DDH awareness, the participants were asked about the DDH description, where the majority of 46.80% answered the unrelated definition, 31.30% selected the correct answer, and 21.80% were unfamiliar with DDH. When our participants were asked at what age they thought a person should be diagnosed with DDH, only 44.20% of participants knew when to diagnose it, while the rest (55.8%) either did not know or thought that delaying the diagnosis after six months is correct. In contrast with study findings in Saudi Arabia in 2021 that found 74% of the participants did not realize that the time of birth is the best time for diagnosis, and just 17% said the best time is after the first month [8].

Infants with more than one DDH risk factor are more likely to develop DDH [12]. This study found that newborns with at least one risk factor have a two-fold higher probability of having DDH than infants without risk factors, and those with several risk factors are more susceptible to developing DDH than those with only one risk factor [12]. A positive family history of DDH, female sex, and breech presentation are the most cited risk factors for DDH. Breech presentation is the largest DDH contributing risk factor [1]. Yet, only non-significant relationships were discovered between oligohydramnios and premature delivery. DDH appears less common in children whose birth weight is less than 2500 g. Also, there was a substantial link discovered between primiparity and DDH [1]. As mentioned above, the breech presentation is the most significant risk factor unknown to 63% of participants. In contrast, 30% found it to be a risk factor. Whereas another study in 2021 reported that 6.4% found that breech presentation is a risk factor [13]. Family history is a risk factor for DDH, yet 58% of our participants stated that they are unfamiliar with that, compared to only 21% who know that it contributes to developing DDH. Similarly, only a minority of Saudi females reported that family history is one of the leading risk factors for DDH [13]. Moreover, 63.80% of our participants were unaware that being a female contributes to developing DDH. Again, many of our participants have no idea that premature birth, high birth baby weight, and oligohydramnios are also risks for DDH. Generally, most participants did not know an answer when we asked about the risk factors, showing that our sample has poor knowledge of DDH risk factors.

In DDH, the treatment goal is for the femoral head to be contained and maintained in the acetabulum by methods ranging from bracing to surgical treatments. The most used treatment algorithm depends on patient age for the treatment approach [2]. As mentioned previously, age is the determining factor for the management method for DDH, answered correctly by 60.9% of the population (Figure 4). Furthermore, most participants have a common misconception that the severity of the disease (65%) and the presence of complications (55.1%) as determining factors for treatment; although they are contributing factors to the treatment, they are not the main determining factor in the treatment approach. In addition, participants were asked about the suitable treatments for children based on 3 age groups (Table 4). The results show a high percentage of misconception in the population regarding which treatment option is indicated according to the patient’s age. Unfortunately, 10.40% believe that DDH is a condition that does not require treatment. At the same time, 21.4% prefer to wait for the child to walk and then obtain the treatment. Early diagnosis and treatment are critical not only to providing the best possible outcomes but also in order to prevent extensive surgeries and complications in the long term [14].

The treatment of DDH is important not only to return to normal anatomy and continue normal development, but untreated cases of DDH will also inevitably face complications. DDH consequences are not limited to childhood; it also reaches adulthood as DDH is the leading cause of osteoarthritis of the hip in adults [2]. Unsurprisingly, 42.70% of participants reported no knowledge about DDH complications. In addition, participants were given a list of six possible complications from which they could choose multiple answers. Many showed great knowledge about DDH complications, the majority (72.3%) identified limping as a complication, and many valid answers were chosen, like limb discrepancy, osteoarthritis, and back pain (54,4%, 43.9%, and 36.9%, respectively). On the other hand, many participants viewed hip pain and inability to walk as a complication of DDH, which were not correct answers. Hip pain was the second most identifiable complication of DDH (54.6%), and the inability to walk represented 41.7% of the answers (Figure 2). Similar findings were seen in a study conducted in Saudi Arabia in 2018; it reveals limping as the most chosen complication, followed by hip pain [8]. The previous study noted a relationship between previous knowledge about DDH and limping as an answer.

Our study suggests that the knowledge level of DDH is poor. The majority had a low knowledge level regarding DDH (72.8%), and only 5.1% had a high knowledge level, which is concerning considering surgical treatment of neglected DDH cases represents 30% of the surgical practice of pediatric orthopedic surgeons in the country [10]. In our study, females had a significantly higher knowledge score than males (p = 0.026), similar to a local study done among pregnant ladies that showed one-third of the participants had good awareness [13]. Education level was also significantly associated with knowledge score toward DDH (p = 0.01); it was observed that the higher the education level, the higher the knowledge score. In our study, the Tukey post-hoc test did not reveal a significant difference when comparing each education level. Alshahrani et al. reported that previous knowledge of DDH among parents had a significant association with parents’ employment and education [8]. In addition, the profession was also significantly associated with knowledge score toward DDH (p < 0.001), and healthcare professionals had a significantly higher knowledge score than non-healthcare professionals.

Similarly, more than half of the primary care physicians in Riyadh (69.2%) had sufficient knowledge of DDH [15]. Also, parents who reported having a child screened for DDH had a significantly higher knowledge score than those who did not have a child screened for DDH (p < 0.001). Also, having a first-degree relative with DDH was significantly associated with a knowledge score toward DDH (p < 0.001), this is similar to a study where 71.4% of the females having a child diagnosed with DDH had good awareness levels [13]. Moreover, the main source of knowledge on DDH was from an affected family member [8].

Since DDH awareness in Saudi Arabia is suboptimal, it is important to educate all community members to effectively address the problem and raise awareness to decrease DDH incidence in Saudi Arabia. This is similar to what happened in Qatar and Japan when they established DDH campaigns that helped decrease its incidence [16]. Therefore, we recommend raising community awareness through awareness campaigns and involving healthcare providers in educating about early intervention and preventing complications. We also recommend that physicians and nurses that supervise children’s health should go through educational programs or seminars for DDH. Moreover, we encourage other researchers to conduct more studies on DDH to assess population awareness, which will assist in lowering the prevalence of DDH in Saudi Arabia.

There are some limitations to our study. It was limited to one geographical location, and there were limited articles about assessing community knowledge about DDH in Saudi Arabia. Our study used an online questionnaire that helped gather responses from different regions in Riyadh. However, this may have contributed to relatively similar results between participants.

## Conclusions

Our study’s findings revealed that most Riyadh residents were unaware of DDH, and their knowledge regarding its risk factors, treatment, and complications was poor. Greater awareness was associated with a family member who was affected by DDH. Additionally, female gender, education level, and healthcare workers have significantly higher levels of awareness about DDH.
